# A Polyphenol-Rich Extract From *Entada abyssinica* Reduces Oxidative Damage in Cryopreserved Ram Semen

**DOI:** 10.3389/fvets.2020.604477

**Published:** 2020-12-02

**Authors:** Mansour Sobeh, Soha A. Hassan, Mahmoud A. E. Hassan, Wael A. Khalil, Mohamed A. O. Abdelfattah, Michael Wink, Abdelaziz Yasri

**Affiliations:** ^1^AgroBioSciences Research Division, Mohammed VI Polytechnic University, Ben-Guerir, Morocco; ^2^Institute of Pharmacy and Molecular Biotechnology, Heidelberg University, Heidelberg, Germany; ^3^Basic Science Department, Faculty of Dentistry, October 6 University, Cairo, Egypt; ^4^Animal Production Research Institute, Giza, Egypt; ^5^Department of Animal Production, Faculty of Agriculture, Mansoura University, Mansoura, Egypt; ^6^College of Engineering and Technology, American University of the Middle East, Kuwait

**Keywords:** *Entada abyssinica*, polyphenolics, semen cryopreservation, sperm ultrastructure, Antioxidant biomarker

## Abstract

The Splinter bean, *Entada abyssinica*, is widely used in folk medicine. In the current work, we profiled the secondary metabolites from *E. abyssinica* bark extract using LC-MS and investigated its effect on cryopreserved ram semen. Twenty-eight compounds, including tannins and gallic acid derivatives that prevailed in the extract, were tentatively identified. Results showed that the quality of the post-thawed semen showed a significant improvement when the extract was added to the extender at a concentration of 375 μg/mL. The progressive motility and plasma membrane integrity of sperm cells were significantly increased in the post-thawed semen; however, the total antioxidant capacity (TAC) was insignificantly increased. A significant decrease in the concentration of hydrogen peroxide was detected as well. No significant changes were observed in activities of lactate dehydrogenase (LDH), alanine aminotransaminase (ALT), and aspartate transaminase (AST) within the treated samples. Intact sperm percentage was significantly increased, while apoptotic and necrotic sperm percentages were reduced significantly. Molecular docking of some individual components from the extract revealed their potential to interfere with the apoptosis cascade in which Bcl-2 is involved. In conclusion, *Entada abyssinica* appears to be useful for cryopreservation presumably owing to its polyphenol content that has potent antioxidant capacity scavenging reactive oxygen species (ROS), enhancing the endogenous antioxidant system and inhibiting lipid peroxidation.

## Introduction

Artificial insemination (AI) is a widely applied technique that uses fresh semen and frozen–thawed sperms. Thus, sperm preservation has many applications in different areas including AI, species conservation, and clinical medicine (1, 2). One of the most growing interests in many countries is ram semen cryopreservation, aiming to increase the quality of productive parameters of the selected flocks. The use of ram frozen–thawed semen eliminates the geographical barriers, helps in preserving endangered breeds, and conserves the biodiversity (3).

The viability of the preserved sperms may be affected by many factors including storage temperature, cryoprotectant concentration, cooling rate, extender composition, free radical contents, seminal plasma contents, and antiseptic factors (4, 5). Scientists are still facing undesirable results of the process plausibly owing to intracellular ice crystal formation, osmotic and chilling damage, which cause sperm cell injury, DNA damage, and cytoplasmic injury (6). Ice crystallization and recrystallization during freezing and thawing techniques induce biochemical and cellular changes and alter the sperm efficiency (7). Sperm motility and morphology may be affected as well by increased membrane permeability after cryopreservation (8, 9). In addition, these post-thawing-induced changes could impair sperm transport and survival inside the female reproductive tract, affecting fertilizing capacity and embryogenesis (8). Therefore, many efforts to maintain and improve sperm viability in these techniques have been developing recently.

The primary antioxidant system in seminal plasma acts as defensive machine by the aid of some enzymes such as glutathione peroxidase, superoxide dismutase, and catalase, which scavenge reactive oxygen species (ROS) resulting from lipid peroxidation (10). This system alone is insufficient to face the oxidative stress during cryopreservation and thawing processes. The dilution of semen during extending causes a decrease in the concentration of the system's antioxidant enzymes leading to strong oxidative stress (11). Moreover, the high content of saturated and polyunsaturated fatty acids in the plasma membrane of ram sperm makes it more susceptible to oxidative stress (12). This emphasizes the importance of supplementing the freezing media with added antioxidants that would minimize the negative effect of ROS and maintain the quality of post-thawed sperm (13). In this regard, different types of supplements are added to the freezing media as protective agents; however, antioxidants of natural origin are of special interest in this concern (14–16).

The Splinter bean, *Entada abyssinica* (Fabaceae), is a widely spread tree in central and eastern tropical Africa. Extracts from *E. abyssinica* bark and leaves have been traditionally used in folk medicine to manage a large number of ailments, such as sleeping sickness, coughs, rheumatic fever, abdominal pain, and diarrhea in west and east Africa (17, 18). A plethora of biological activities were documented for different plant parts. For instance, a complex extract from the bark containing alkaloids, diterpenoids, saponins, and flavonoids exhibited antimicrobial, antifungal, and antiviral activities as well as cytotoxic properties against drug-resistant cancer cells (19, 20). For the leaves, anti-inflammatory and antioxidant activities among other various beneficial effects were also described (21).

In this work, we characterized the chemical constituents of a methanol extract from *E. abyssinica* bark utilizing HPLC-MS/MS. The potential antioxidant activities of the extract were evaluated *in vitro*. We investigated the cryopreservative and antioxidative effects of adding the extract to semen extender on the quality of Ossimi ram (*Ovis aries*) semen. Sperm vitality and morphology were investigated in detail. Sperm ultrastructure was also evaluated after the thawing process. Oxidative biomarkers and enzymatic activities in the post-thawed extender were studied. Moreover, the major identified compounds in the extract were docked to the Bcl-2:BH3 interface to evaluate their individual antiapoptotic potential.

## Materials and Methods

### Plant Material and Extraction

*Entada abyssinica* bark material was collected from trees growing in Lupaga Site in Shinyanga, Tanzania, and kept under accession number P7301, at IPMB, Heidelberg University. The bark sample was ground and extracted with 100% methanol at ambient temperature for 3 days (6 × 500 mL). The filtered extracts were evaporated under reduced pressure at 40°C. The frozen residue was lyophilized yielding 25% extraction yield based on the initial dry weight.

### *In vitro* Antioxidant Activities

2,2-Diphenyl-1-picrylhydrazyl (DPPH), ferric-reducing ability (FRAP), and total phenolic content assays were done according to Sobeh et al. (22). All assays were done in triplicates.

### HPLC-PDA-ESI-MS/MS

The chemical constituents of the bark extract were annotated utilizing a ThermoFinnigan LCQ-Duo ion trap mass spectrometer (ThermoElectron Corporation, Waltham, MA, USA) with an ESI source (ThermoQuest Corporation, Austin, TX, USA) as detailed in Sobeh et al. (22).

### Ethical Approval

The semen samples were collected from a sheep flock belonging to the Animal Production Research Station, El-Karada, Kafrelsheikh, Animal Production Research Institute (APRI), Agricultural Research Center, Ministry of Agriculture, Egypt, in cooperation with the Physiology and Biotechnology Laboratory, Animal Production Department, Faculty of Agriculture, Mansoura University, Egypt. This study was approved by the Ethical Committee of Mansoura University.

### Animal Management

Five sexually mature and clinically healthy Ossimi rams (60–80 kg LBW and 2–4 years old) were trained to serve an artificial vagina for collection of semen ejaculates. The animals were kept under natural photoperiod in open shaded stockyard, raised under the same environmental conditions, and fed on concentrate feed mixture with free access to trace mineralized salt lick blocks and drinking water all time.

### Collection of Semen

Ejaculates were collected as per conventional artificial vaginal method once weekly from each ram for 5 weeks before feeding at 7–8 a.m. A total of 25 ejaculates were transferred immediately to a water bath at 37°C. Only ejaculates with overall motility ≥70% and minimum sperm concentration ≥2.2 × 10^9^ sperm cells/mL were selected for the experiment, pooled, and then divided into five aliquots to be subjected to the different experimental treatments.

### Preparation of Semen Extenders

Tris-citric-soybean lecithin extender (TSBL) was used in this study as a control. It is composed of 250 mM Tris (AppliChem, Germany), 87.5 mM citric acid monohydrate (AppliChem, Germany), 69 mM glucose (Sigma Aldrich, USA), 1% (w/v) soybean lecithin (L-a-phosphatidyl choline, LAB: product number MC041), 5% (v/v) glycerol, 100 IU/mL of penicillin, and 100 μg/mL of streptomycin. The extender was shaken gently and warmed in a water bath up to 37°C before use. Osmolarity level and pH value were adjusted to 300 mOsmol and pH 7.3, respectively, before the addition of cryoprotectants.

### Biocompatibility of the Extract

The compatibility of the extract with the ram semen was investigated by evaluating the sperm characteristics (progressive motility, vitality, abnormality, membrane integrity, and acrosome integrity) after diluting the fresh semen with different concentrations of the extract and equilibrating for 4 h at 5°C before cryopreservation.

### Semen Freezing and Thawing

Dilution of the collected semen was carried out at 37°C with a ratio of 1:10 (semen:extender). Final sperm concentrations were adjusted to 220 × 10^6^ sperm/mL. Extended semen was cooled gradually to 5°C for 4 h, and then the equilibrated semen was aspirated into 0.25 mL French straws and sealed (IMV technologies, France). The straws were exposed to liquid nitrogen vapors for 10 min and finally placed into liquid nitrogen at −196°C. The straws remained in liquid nitrogen until thawing at 37°C in a water bath for 30 s.

### Experimental Design

Using supplemented TSBL extender, the semen was extended by adding different concentrations of the bark extract (0, 125, 250, 375, and 500 μg/mL extender) before cryopreservation in liquid nitrogen.

### Semen Evaluation

#### Sperm Progressive Motility

The percentage of progressive sperm motility, which was defined as the ability of a spermatozoa to move forward in a long semi-arc pattern, was determined to analyze the sperm motility. An aliquot (10 μL) of diluted semen was mounted on a previously warmed slide, then covered and investigated by phase-contrast microscope (DM 500, Leica, Switzerland) supplied with a hot stage at 37°C at 100x magnification. A total number of 200 spermatozoa per slide were counted, and the analysis was conducted in three replicates.

#### Sperm Vitality

Sperm vitality was investigated in a smear of semen stained with 5% eosin (vital stain) and 10% nigrosin (background stain) to estimate alive and dead sperm cells according to Moskovtsev and Librach (23). Percentage of alive sperm cells (unstained ones) was calculated for 300 sperm cells per sample and examined under light microscope at magnification (400x).

#### Morphological Sperm Abnormalities

Abnormalities were assessed in 300 sperm cells during vitality test using a light microscope. The following criteria were considered: (i) abnormal tails (coiled tail, broken tail, terminally coiled tail, double tail), (ii) abnormal heads (microcephalic head, pear shaped head, round short head, loose head, double head), and (iii) cytoplasmic droplets proximal and distal droplets according to Aamdal et al. (24).

#### Plasma Membrane Integrity

Plasma membrane integrity of spermatozoa was assessed using hypo-osmotic swelling test (HOS-t) according to the protocol described by Neild et al. (25). Briefly, 50 μL of semen was incubated for 30 min at 37°C in a hypo-osmotic solution (500 μL at osmolarity level of 75 mOsm/kg H_2_O), containing fructose (6.75 g/L) and sodium citrate (3.67 g/L). A sample of the mixture was placed on a slide and covered with a cover slip. Sperm cells showing coiled or swollen tails (with functional intact membranes) were counted in all samples using phase-contrast microscope (400x) within total count of 300 sperm cells per slide.

#### Acrosome Integrity

A drop of frozen–thawed semen was placed on a pre-warmed glass slide and allowed to air-dry. The slide was then fixed in 5% formaldehyde for 30 min, washed afterward under running water, dried, and then immersed in a Giemsa solution for 3 h at 37°C. Finally, the slides were washed under running tap water before dried. All slides were investigated under phase-contrast microscope using oil immersion lens with 200 sperm cells counted.

#### Biochemicals Assay in the Extender After Thawing

The following parameters in seminal extender were evaluated using the available commercial kits (Biodiagnostic, Egypt) according to the manufacturers' instructions and the mentioned biochemical methods. Total antioxidant concentration (26), hydrogen peroxide, H_2_O_2_ (27), enzymatic activity of lactic dehydrogenase (LDH) (28), alanine transaminase (ALT), and aspartate transaminase (AST) (29) were noted. The tested parameters were measured using a spectrophotometer (Spectro UV-VIS Auto, UV-2602, Labomed, USA).

#### Ultrastructure Changes by Transmission Electron Microscope

Semen samples were prepared for transmission electron microscope (TEM) as per the method described by Oliveira et al. (30) with some modifications. In brief, semen extender samples (500 μl) were centrifuged and suspended in 2.5% glutaraldehyde in phosphate-buffered saline for 2 h at 4°C to allow for first fixation. Washing the post-fixed samples was carried out by 1% osmium tetroxide for 90 min at room temperature and followed by dehydration through ascending grades of ethanol. The dehydrated samples were treated with propylene oxide, infiltrated in an equal mixture of epon: propylene oxide, and finally embedded in Epon resin (Epon 812; FlukaChemie, Switzerland). Specimens were transferred into polyethylene capsules using toothpick then placed in an oven for polymerization at 60°C for 24 h. Ultrathin-sections (60–70 nm) were cut using an ultramicrotome. Observation of the obtained sections was done using a JEOL-JEM 2100 TEM operated at 80 KV. The ultrastructure of sperm was examined in 300 sperms per sample. The observed results were categorized into three patterns and defined according to sperm criteria as described by Baccetti et al. (31): (i) Intact spermatozoa: The ultra-structure of all sperm components (plasma membrane, acrosome, nucleus, and cytoplasm) is normal with no defects. (ii) Apoptotic sperm: altered nuclear structure with irregular marginated chromatin, cytoplasmic residue and binucleate and multinucleate sperm. Discontinuous or deformed plasma membrane or deformed acrosomal structure. (iii) Necrotic spermatozoa: distorted nuclear structure with necrotic chromatin and cytoplasmic residue. Broken or discontinuous plasma membrane deformity or acrosomal absence.

### Molecular Docking

The *in silico* molecular docking computational tool was applied to evaluate, on a molecular level, the antiapoptotic potential of the major compounds identified in *Entada abyssinica* bark extract. The X-ray crystallographic structure of the Bcl-2:BH3 interface complex (PDB code: 4B4S) was downloaded from the Protein Data Bank (www.rcsb.org). The docking protocol was applied using Molecular Operating Environment (2010.10; Chemical Computing Group Inc., Montreal, Canada) software. Downloaded protein was protonated to add the hydrogen atoms that were not detected during the crystallization process. Chemical structure of the compounds selected for docking was downloaded directly from PubChem database or drawn using the MOE builder tool. Compounds were then washed to set their ionization state. The MMFF94x force field was used to do the energy minimization for the compounds. Docking was done applying the default settings of placement and scoring.

### Statistical Analysis

The general linear model analysis of variance (ANOVA) was applied for data statistical analysis using SAS software (32). Different concentrations of the bark extract were statically tested for their effect. Tukey's test was applied to examine the significant differences among treatments for all considered parameters. Arcsine transformation was performed before the analysis of variance for all percentage values.

## Results

### Chemical Profiling of the Bark Extract

Altogether, 28 secondary metabolites were tentatively identified in the methanol extract from *E. abyssinica* bark based on their molecular weight, mass fragmentation pattern, available authentic compounds, in-house library, and online literature. Tannins and gallic acid derivatives dominated the extract (Table 1 and Supplementary Figure 1). Among the annotated compounds, one showed a signal at 37.86 min and demonstrated a molecular ion peak at [M – H]^−^ *m/z* 521 with three daughter ions at 331, 271, 169; it was tentatively characterized as dimethyl caffeoyl galloylglucose (Supplementary Figure 2). Another compound exhibiting [M – H]^−^ *m/z* at 585 and three main ions at 331, 271, 169, was tentatively annotated as *p*-coumaroyl pyrogalloylgalloylglucose (Supplementary Figure 3). Additionally, a signal with [M – H]^−^ *m/z* 461 was identified as cinnamoyl-*O*-galloylglucose (Supplementary Figure 4).

**Table 1:** Phytochemical profiling of *Entada abyssinica* bark extract.

**No**.	**RT**	**M-H^**−**^**	**MS/MS**	**Tentatively identified compounds**
1	1.53	133	115	Malic acid^a^
2	2.37	417	153, 241, 285	Gentisic acid dipentoside
3	3.18	609	179, 305, 441	(epi)Gallocatechin-(epi)gallocatechin
4	5.25	593	289, 407, 425	(epi)Catechin-(epi)gallocatechin^a^
5	6.26	305	179, 221, 287	(epi)Gallocatechin^a^
6	10.97	761	305, 423, 609	(epi)Gallocatechin-(epi)gallocatechin gallate
7	11.50	183	125, 169, 183	Methylgallate
8	12.19	483	169, 271, 331	Digalloyl glucose
9	13.05	289	179, 205, 245	(epi)Catechin^a^
10	17.47	457	179, 305	Gallocatechin gallate^a^
11	18.17	745	289, 407, 593	(epi)Catechin-(epi)gallocatechin gallate^a^
12	18.98	457	169, 305, 331	(epi)Gallocatechin gallate^a^
13	20.17	729	289, 559, 577	(epi)Catechin-(epi)catechin gallate^a^
14	20.65	457	179, 305	Gallocatechin gallate
15	21.78	617	285, 493, 599	Kaempferol syringyl gallate
16	22.60	729	289, 559, 577	(epi)Catechin-(epi)catechin gallate
17	25.19	541	169, 211, 271, 541	Hydroxybenzoylbenzyl-*O*-galloyl-glucoside
18	26.35	615	271, 301, 463	Quercetin galloylglucose
19	27.73	441	179, 245, 289	Catechin gallate
20	28.73	441	179, 245, 289	(epi)Catechin gallate^a^
21	30.34	477	169, 315, 331, 417	Coumaroyl-*O*-galloylglucose
22	32.19	507	169, 323, 445, 447	Methyl gallate caffeoylglucose
23	34.12	601	169, 313, 439	Caffeoyl pyrogallol galloylglucose
24	36.38	435	273	Phlorizin
25	37.86	521	169, 271, 331	Dimethyl caffeoyl galloylglucose
26	39.10	585	169, 313, 439	*p*-Coumaroyl pyrogalloylgalloylglucose
27	41.88	477	169, 313, 327	Coumaroyl-*O*-galloylglucose
28	43.45	461	169, 313, 401	Cinnamoyl-*O*-galloylglucose

a *Identified according to Sobeh et al. (22)*.

### Antioxidant Activity of the Bark Extract and Its Effect on Post-thawed Semen Extender of *Ovis aries*

To initially investigate the antioxidant potential of the extract, DPPH and FRAP assays were performed, and TPC was determined. The extract demonstrated substantial activity in both assays and showed an appreciable TPC of 240 mg GAE/gm extract (Table 2).

**Table 2:** Antioxidant activity of the methanol bark extract of *Entada abyssinica*.

	**DPPH**	**FRAP**	**TPC**
**Sample**	**(EC_**50**_ μg/mL)**	**(mM FeSO_**4**_ equivalent/mg sample)**	**mg GAE/g extract**
Bark extract	35.8	13.21	240
Ascorbic acid	2.92 ± 0.29	–	–
Quercetin	–	24.04 ± 1.23	–

*Ascorbic acid and quercetin are positive controls. DPPH, 2,2-diphenyl-1-picryl-hydrazyl-hydrate; FRAP, ferric-reducing antioxidant power assay; TPC, total phenolic content*.

The compatibility of the extract with the fresh ram semen was investigated. The extract was found to be safe in concentrations up to 500 μg/mL. Progressive motility, vitality, abnormality, and acrosome integrity were not affected (Table 3). Then after, the potential value of the extract as a supplement during cryopreservation was investigated through examining the characteristics, morphological abnormalities, the oxidative stress biomarkers, and the ultrastructural changes in the post-thawed ram sperms. The extract significantly enhanced the progressive motility and membrane integrity (Table 4). However, other parameters including sperm vitality, abnormal morphology, and acrosome integrity were not significantly affected when compared to the control.

**Table 3:** Sperm characteristics in extender of post equilibrated (at 5°C for 4 h) fresh ram semen supplemented with different concentrations of the bark extract (means ± SE, *n* = 5).

**Sample**	**Progressive motility**	**Vitality**	**Abnormality**	**Membrane integrity**	**Acrosome integrity**
	**%**				
Control	81.0, 1.87	77.6, 3.50	7.6, 1.03	75.4, 2.42^b^	92.4, 0.51
Extract 125 μg/mL	84.0, 1.87	82.6, 1.60	8.4, 0.51	81.2, 2.01^b^	93.4, 1.33
Extract 250 μg/mL	84.0, 1.87	79.6, 1.63	10.2, 1.46	82.8, 1.59^b^	94.0, 1.30
Extract 375 μg/mL	86.0, 1.87	82.8, 1.85	7.8, 0.49	84.0, 1.70^b^	93.0, 0.45
Extract 500 μg/mL	80.0, 2.24	77.6, 1.44	10.4, 1.44	78.2, 1.53^b^	94.0, 0.71

a, b *Means denoted within the same column with different superscripts are significantly different at p < 0.05 compared with the control group*.

**Table 4:** Sperm characteristics in extender of post-thawed ram semen supplemented with different concentrations of the bark extract (means ± SE, *n* = 5).

**Sample**	**Progressive motility**	**Vitality**	**Membrane integrity**	**Acrosome integrity**	**Abnormal morphology**
	**%**				
Control	48.0, 2.55^b^	44.6, 3.63	43.4, 2.80^b^	88.2, 0.86	12.4, 0.51
Extract 125 μg/mL	56.0, 1.87^a^	48.6, 2.34	45.6, 2.16^b^	88.8, 0.58	12.8, 0.97
Extract 250 μg/mL	56.0, 2.92^a^	48.8, 1.91	45.4, 2.36^b^	88.0, 1.73	14.4, 2.01
Extract 375 μg/mL	59.0, 1.87^a^	51.0, 1.90	53.6, 2.87^a^	86.8, 0.80	15.6, 1.29
Extract 500 μg/mL	52.0, 2.00^ab^	45.8, 2.08	41.2, 2.03^b^	88.0, 0.84	14.8, 0.97

a, b *Means denoted within the same column with different superscripts are significantly different at p < 0.05*.

In sperm-based cell assays, the total antioxidant capacity (TAC) was increased insignificantly at a concentration of 375 μg/mL, while the concentration of H_2_O_2_ was significantly reduced when compared to the control group without significant change in the concentration of LDH, AST, and ALT activities (Table 5).

**Table 5:** Antioxidant capacity, oxidative stress, and enzymatic activity in extender of post-thawed ram semen supplemented with different concentrations of the bark extract (Means ± SE, *n* = 3).

**Sample**	**TAC**	**H_**2**_O_**2**_**	**LDH**	**AST**	**ALT**
	**Mm/L**	**nm/L**	**U/mL**	**U/L**
Control	0.20, 0.07	0.36, 0.05^ab^	147.5, 24.84	67.3, 3.33	18.7, 0.67
Extract 125 μg/mL	0.31, 0.05	0.27, 0.01^bc^	139.4, 38.17	52.7, 6.36	15.3, 2.40
Extract 250 μg/mL	0.36, 0.03	0.27, 0.03^bc^	143.9, 19.48	48.7, 7.69	16.0, 1.15
Extract 375 μg/mL	0.45, 0.03	0.2, 0.06^c^	134.0, 10.94	49.3, 9.33	16.7, 1.76
Extract 500 μg/mL	0.30, 0.09	0.45, 0.05^a^	118.7, 6.79	68.0, 4.00	16.0, 2.31

a, b, c *Means denoted within the same column with different superscripts are significantly different at p < 0.05*.

### Effect of the Bark Extract on Sperm Ultrastructure Post-thawing

**Figure 1:**
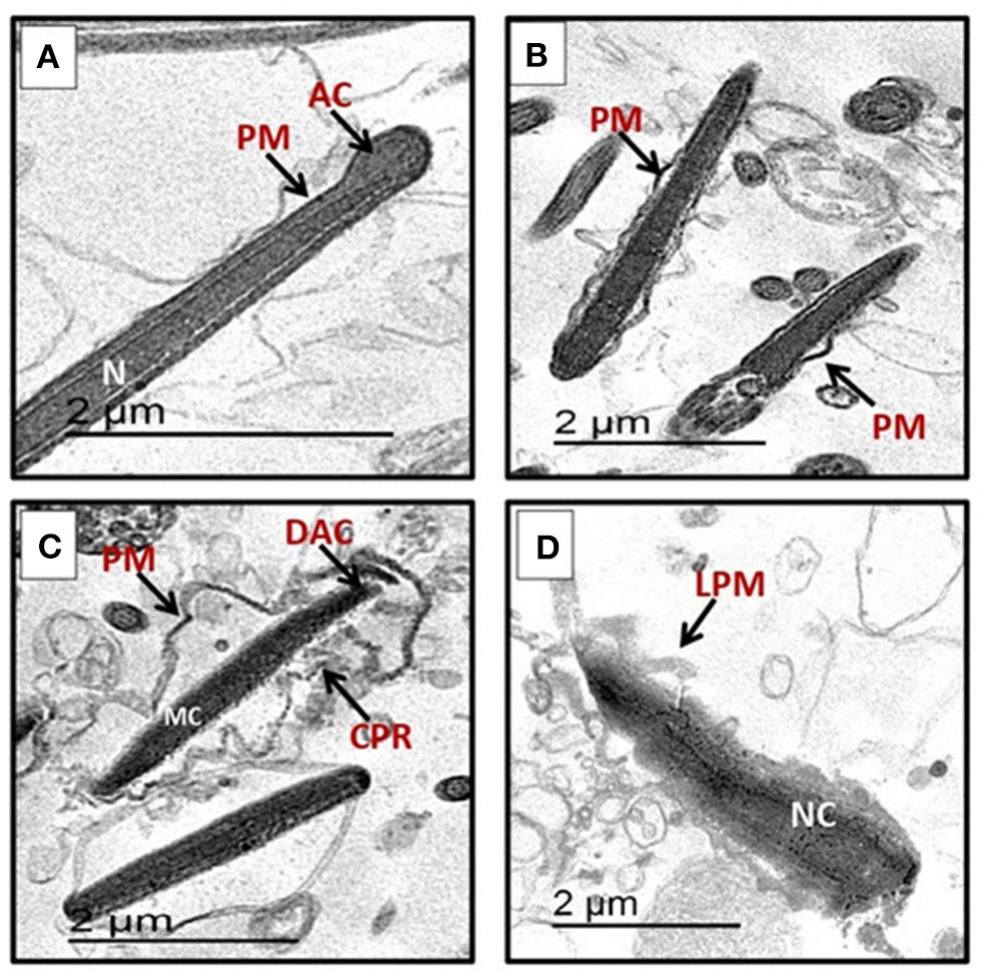
Transmission electron microscope (TEM) micrographs of ram spermatozoa in post-thawed semen showing **(A)** normal sperm with complete nuclei (N), homogenous condensed chromatin, intact acrosomal cap (AC), normal plasma membrane (PM). **(B)** Early apoptotic sperm cells with mildly swollen plasma membrane (PM). **(C)** Sperm with apoptotic nucleus characterized by marginated chromatin (MC), cytoplasmic residue (CPR), broken plasma membrane (PM), detached with apical ridge formed near the tip of acrosomal cap (DAC), degenerated acrosome. **(D)** Sperm with necrotic chromatin (NC), with cytoplasmic residue (CPR), lost plasma membrane (LPM), and degenerated acrosome (DAC).

The sperm characterization in different groups was assessed based on the criteria mentioned in the Materials and Methods section. The normal sperms appear intact with continuous plasma membrane (PM) along the acrosomal cap (AC), intermediate, and tail regions as seen in Figure 1A. Normal structure of dense elongated nucleus was also noticed. The abnormal sperms in different groups show dented plasma membrane (PM) with gaps observed between membrane and nucleus (Figure 1B). Figures 1C,D show notable abnormalities in sperm membrane system exhibited as cytoplasmic residues (CPR), destruction in acrosomal region (DAC), and complete lysis of plasma membrane (LPM). At the level of 375 μg/mL, the extract significantly increased the percentages of intact sperm cells and significantly decreased apoptotic spermatozoa without significant change in necrotic spermatozoa when compared to the control (Table 6).

**Table 6:** Percentage of sperm groups characterized using TEM in extender of post-thawed ram semen supplemented with different concentrations of the bark extract (Means ± SE, *n* = 3).

**Sample**	**Intact sperm**	**Apoptotic sperm**	**Necrotic sperm**
	**%**		
Control	38.3 ± 2.03^b^	40.3 ± 0.88^b^	21.3 ± 1.86^c^
Extract 125 μg/mL	48.0 ± 2.08^a^	31.7 ± 1.86^c^	21.7 ± 2.03^c^
Extract 250 μg/mL	41.7 ± 0.88^b^	30.3 ± 0.88^c^	28.0 ± 0.58^ab^
Extract 375 μg/mL	53.3 ± 1.45^a^	21.7 ± 1.45^d^	25.0 ± 1.73^bc^
Extract 500 μg/mL	16.3 ± 2.03^c^	53.3 ± 2.19^a^	30.3 ± 0.67^a^

a, b, c, d *Means denoted within the same column with different superscripts are significantly different at p < 0.05*.

### Molecular Docking Study

The major compounds identified in the extract were docked to the surface interface of the Bcl-2:BH3 complex. As shown in Table 7, the docked compounds showed appreciable free binding energy manifested by the low value of the docking scores. This reveals the potential of the extract components to inhibit the dimerization of the Bcl-2 with the BH3 domain of the proapoptotic Bim protein, thus hindering the apoptosis cascade.

**Table 7:** Docking scores of the docking poses obtained upon docking major compounds identified in the bark extract to Bcl-2:BH3 complex interface.

**Compound number**	**Compound name**	**Docking score (kcal/mol)**
5	Epigallocatechin	−13.16
6	Epigallocatechin-epigallocatechin gallate	−16.49
8	Digalloyl glucose	−12.72
12	Epigallocatechin gallate	−15.69
13	Epicatechin-epicatechin gallate	−16.66
18	Quercetin galloylglucose	−13.62
20	Epicatechin gallate	−13.89
24	Phlorizin	−9.44
25	Dimethyl caffeoyl galloylglucose	−13.57
26	*p*-Coumaroyl pyrogalloyl galloylglucose	−13.23
27	*p*-Coumaroyl galloylglucose	−13.12
28	Cinnamoyl galloylglucose	−10.32

## Discussion

Previous studies reported that sperm viability and motility, the integrity of both plasma membrane and acrosome in post-thawed semen are negatively affected during the cryopreservation process (33, 34). The mechanism by which the cryopreservation affects motility has not been fully explained; however, a solid correlation links the percentage of immotile spermatozoa and mitochondrial defects in post-thawing sperm (33). Sperm cells have a large surface with small size; thus, they are sensitive to the damage caused by cryopreservation and the consequences of ROS production (35). In this study, the H_2_O_2_ concentration was significantly decreased in the sperm extender upon adding the plant extract in increasing concentrations (125, 250, 375, and 500 μg/mL). It was noticeable that the higher the extract concentration used, the stronger the antioxidant effect until a concentration of 375 μg/mL.

Reactive oxygen species (ROS) may cause apoptosis and DNA damage plus other cellular alterations such as lipid peroxidation, disruption of plasma membrane, and mitochondria (36). The observed significant improvement of the sperms' membrane integrity and progressive motility in the group supplemented with the extract (375 μg/mL) could be explained by the extract's substantial antioxidant potential that counters the effect of oxidative stress produced by cryopreservation. By increasing the concentration of the extract to 500 μg/mL, the post-thawed sperm quality started to decline significantly compared to the other concentrations. This may be due to the high concentration of tannins that potentially inhibit the activity associated with apoptosis regulation (37). Our findings are in agreement with previous reports that recommend the addition of different antioxidants in semen extenders during cryopreservation (13, 14, 38, 39).

Cryopreservation induces negative changes in plasma membrane and acrosomal structure (14). In the current study, the percentage of the intact sperms was significantly increased upon adding the extract to the extender in a dose-dependent manner until a concentration of 500 μg/mL. On the contrary, the percentage of the apoptotic sperms was significantly decreased in the groups where the extract was added in the concentrations 125, 250, and 375 μg/mL. The last group (500 μg/mL) recorded the highest value of apoptotic sperms.

Plasma membrane defects may impair the sperm vitality and motility (40). The decreased motility of the preserved spermatozoa was reported to be as a result of the ultrastructural changes taking place during the process (41). The physical and chemical factors to which a sperm is exposed are the main causes of such alterations. Ice crystals formation around the cell membranes and increasing of the permeability are the most probable reasons. Formation of ROS, on the other side, affects plasma membrane integrity, nuclear structure, and leads to apoptosis (42). Previous reports described the antioxidant effect of caffeic acid and its derivatives on normal cells. Epigallocatechin 3-gallate (EGCG) has been studied *in vitro* and *in vivo*. It was reported that EGCG scavenges hydroxyl radicals that react with plasma membrane phospholipids and proteins, which, in turn, improves DNA fragmentation (43). Epicatechin, gallocatechin gallate, and quercetin galloylglucose, secondary metabolites identified in the extract, exhibited similar activities (14, 44).

**Figure 2:**
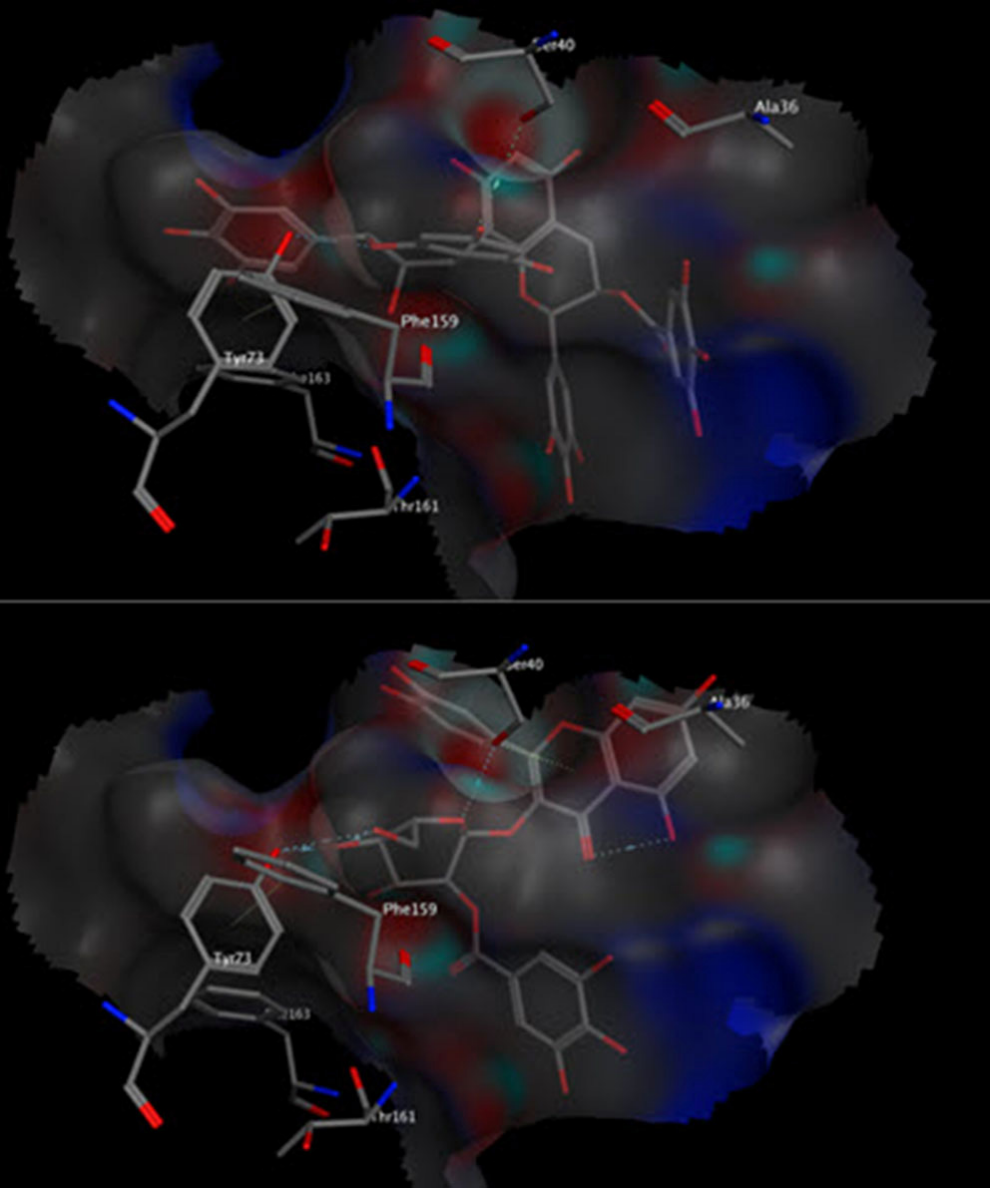
Three-dimensional poses of compounds 6 (top) and 18 (bottom) docked into the Bcl-2:BH3 surface interface.

Molecular modeling was conducted to gain more insights about the antiapoptotic potential of the extract. The major identified compounds were docked into the surface interface of Bcl-2:BH3 complex. It is reported that the programmed cell death (apoptosis) is regulated by the family of B-cell lymphoma-2 proteins (Bcl-2), which comprises antiapoptotic proteins such as Bcl-2 and proapoptotic proteins such as Bim (45). It is well accepted that apoptotic pathways are activated through heterodimerization between pro- and antiapoptotic members into a protein complex such as that of Bcl-2 and Bim. The BH3 domain of the proapoptotic Bim protein binds to a hydrophobic cleft on the Bcl-2 surface initiating the apoptotic pathways (46). Interfering with such interaction by small organic molecules could hinder such interaction and prohibit cell death. The docked compounds were able to bind successfully to the Bcl-2:BH3 interface with appreciable binding energies affording several polar and non-polar interactions with the amino acid residues in the binding site. Compounds 6, 18, 24, 25, and 27 were even able to interact with the Tyr73 residue, which was reported to be among the residues contributing favorably to the binding energy of the Bcl-2:BH3 complex (47). Out of these compounds, epigallocatechin–epigallocatechin gallate (6) and quercetin galloylglucose (18) showed the minimal binding energy of 16.49 and 13.62 kcal/mol, respectively (Figure 2). Because polyphenols are partially ionized in physiological pH, we considered the docking of the ionized form of the compounds. They showed comparable docking scores with some additional ionic interactions with the basic amino acid residues in the binding site. Our results are in agreement with other studies that have reported antiapoptotic activity for tannin-rich extracts such as *Lannea stuhlmannii, Lannea humilis*, and *Senna sengueana* (22, 48).

## Conclusion

The present study profiled the chemical composition of *Entada abyssinica* bark extract. In addition, it highlights the antioxidant activities of the extract *in vitro* and in a semen-based model. Taken together, the obtained results suggest that *Entada abyssinica* extract could be useful as a natural antioxidant that have a potential activity to protect cryopreserved sperm cells against oxidative stress. Nevertheless, the ability of the extract to attain higher fertilization rates in reproductive technologies is recommended to be studied in more detail.

## Data Availability Statement

The original contributions presented in the study are included in the article/Supplementary Materials, further inquiries can be directed to the corresponding author/s.

## Ethics Statement

The animal study was reviewed and approved by the Ethical Committee of the Mansoura University.

## Author Contributions

MS took part in the conceptualization, methodology, software, data curation, writing, review, and editing, and visualization. SH took part in the conceptualization, methodology, data curation, writing the original draft, and visualization. MH took part in the methodology, software, writing the original draft, and writing, review, and editing. WK took part in the conceptualization, methodology, formal analysis, data curation, and writing the original draft, reviewing, and Editing. MA was in charge of the methodology, formal analysis, data curation, and writing the original draft. MW was involved in the conceptualization, writing, reviewing, and editing, and project administration. AY took part by writing, reviewing, and editing and by project administration. All authors contributed to the article and approved the submitted version.

## Conflict of Interest

The authors declare that the research was conducted in the absence of any commercial or financial relationships that could be construed as a potential conflict of interest.

## Supplementary Material

Supplementary Figure 1 LC-MS profile of *Entada abyssinica* bark extract.

Supplementary Figure 2 **(A)** MS/MS profile of dimethyl caffeoyl galloylglucose. **(B)** Proposed fragmentation pattern of compound 25.

Supplementary Figure 3 **(A)** MS/MS profile of *p*-coumaroyl pyrogalloylgalloylglucose. **(B)** Proposed fragmentation pattern of compound 26.

Supplementary Figure 4 MS/MS profile of cinnamoyl-*O*-galloylglucose (compound 28).
