# Supplementary material for: A Polyphenol-Rich Extract From Entada abyssinica Reduces Oxidative Damage in Cryopreserved Ram Semen
Source: Front Vet Sci. 2020 Dec 2;7:604477. doi: 10.3389/fvets.2020.604477 (PMC7740001; doi:10.3389/fvets.2020.604477)
Supplement: Supplementary Figure 1 — LC-MS profile of Entada abyssinica bark extract. [file Data_Sheet_1.docx]

Supplementary Material

## Supplementary Figures

##
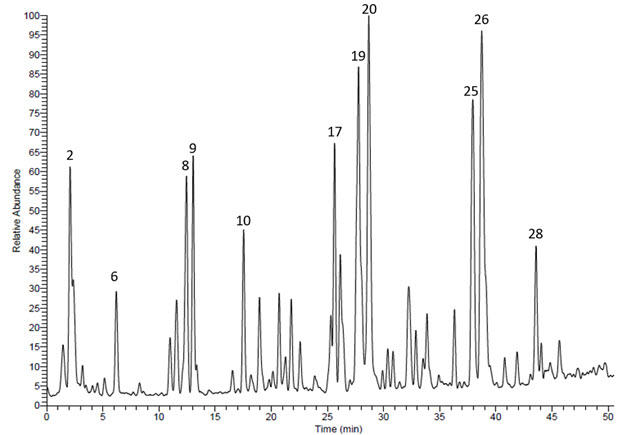


## Figure 1S. LC-MS profile of *Entada abyssinica* bark extract.


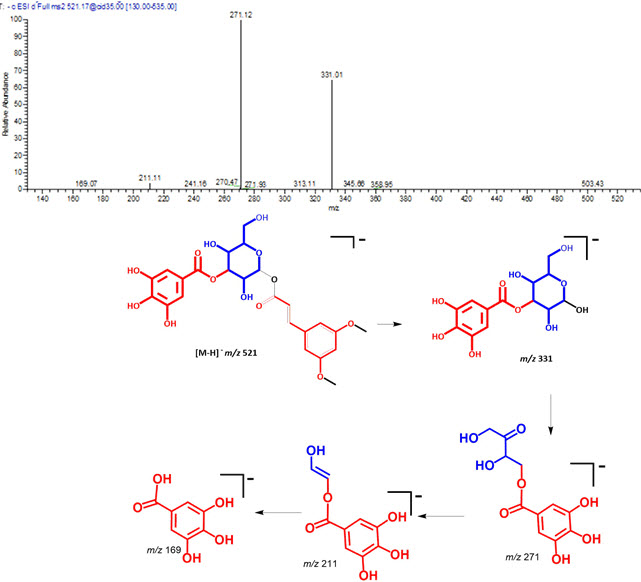
**Figure 2S.** (a) MS/MS profile of dimethyl caffeoylgalloylglucose. (b) Proposed fragmentation pattern of compound 25.

**
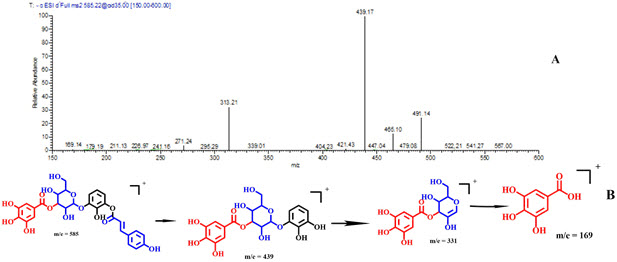
Figure 3S.** (a) MS/MS profile of *p*-coumaroylpyrogalloylgalloylglucose. (b) Proposed fragmentation pattern of compound 26.


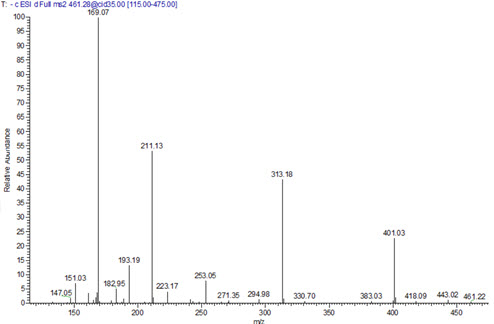


**Figure 4S.** (**a**) MS/MS profile of cinnamoyl-*O*-galloylglucose (compound 28)
